# Impact of aortic morphology on VA-ECMO performance: a combined statistical shape modeling and CFD approach

**DOI:** 10.3389/fbioe.2025.1620897

**Published:** 2025-07-03

**Authors:** Marilena Mazzoli, Emanuele Gasparotti, Emanuele Vignali, Dorela Haxhiademi, Simona Celi

**Affiliations:** ^1^ BioCardioLab, Bioengineering Unit, Fondazione Monasterio, Massa, Italy; ^2^ Department of Information Engineering, University of Pisa, Pisa, Italy; ^3^ Anesthesia and Intensive Care Unit, Fondazione Monasterio, Massa, Italy

**Keywords:** VA ECMO, computational fluid dynamics, statistical shape model, aorta, watershed zone

## Abstract

**Introduction:**

The veno-arterial extracorporeal membrane oxygenation (VA ECMO) system is a temporary support procedure to provide oxygenated blood in patients with cardiac or respiratory failure. Despite being widely used in clinical reality, the VA ECMO has some well-known flaws, like the formation of a 'watershed zone' as a consequence of the mixing of native blood from the left ventricle and retrograde oxygenated blood from the ECMO pump. A deeper knowledge of the watershed zone behavior could contribute to better defining the oxygenation balancing for the patient.

**Methods:**

From this concept, this study proposes a numerical approach combined with Statistical Shape Modeling (SSM) to evaluate the effect of aortic morphology variations on the different conditions of VA ECMO support. In particular, a new SSM approach was adopted to define 48 synthetic aortic morphologies starting from patient-specific computed tomography datasets. After assessing the morphological differences, the first 10 modes were selected to generate volumetric meshes for a Computational Fluid Dynamics (CFD) analysis. A total of 20 cases were simulated in the healthy conditions, while four cases were simulated with a 70% reduction of cardiac output and three different levels of ECMO support (0, 4, and 6 L/min).

**Results:**

From the simulated results, it was possible to assess the behavior of the watershed zone as a function of aortic morphological parameters and ECMO level.

**Discussion:**

The results confirmed the significant effect of ECMO level on the position and distribution of the zone.

## 1 Introduction

Cardiogenic shock (CS) is a pathological condition most commonly caused by myocardial infarction, leading to a significant reduction in cardiac output (CO) due to myocardial cell death (Vrints et al., 2024). As CO decreases, the left ventricle (LV) becomes unable to supply sufficient oxygenated blood to the systemic circulation, resulting in life-threatening consequences if not promptly managed. Several mechanical support systems exist to temporarily maintain circulation and oxygenation in patients with CS (Basir et al., 2023). Among these, veno-arterial extracorporeal membrane oxygenation (VA ECMO) is a widely adopted technique (Banfi et al., 2016). VA ECMO operates by draining venous blood through a first drainage cannula, passing it through an oxygenator with a rotary pump, and returning the oxygenated blood to the arterial circulation via a second return cannula. This process provides systemic oxygenation and supports LV function. Various cannulation strategies exist for the arterial return (Pavlushkov et al., 2017). A commonly adopted approach foresees an iliac artery percutaneous cannulation (Napp et al., 2016). In this configuration, ECMO flow is retrograde relative to native LV flow.

The VA ECMO appears to be a very successful procedure on the basis of its widespread use for the management of CS cases. Nevertheless, some pitfalls and flaws are well known to physicians. The process of VA ECMO management requires a significant level of caution and clinical experience. This aspect is underlined by the fact that, for example, there are no universally standardized protocols for VA ECMO weaning, and every individual center follows its own protocols (Tsiouris et al., 2023). One critical consideration is the increased afterload on the LV due to ECMO flow, which can exacerbate myocardial strain (Singh et al., 2020). Additional complications, including ischemia and thromboembolic events affecting the intestines, kidneys, and brain, are also reported, though their exact mechanisms remain not fully understood (Shen et al., 2022). A phenomenon frequently observed in clinical practice is the formation of a mixing region, known as the watershed zone, between the blood ejected by the LV and the blood delivered by the ECMO pump (Prisco et al., 2022). This watershed zone arises due to the retrograde nature of VA ECMO flow when peripheral cannulation is used. The behavior of this mixing zone is strongly linked to differential hypoxemia, a complication of VA ECMO in which upper and lower body regions receive blood with differing oxygenation levels (Rozencwajg et al., 2023). Furthermore, the complex formation of this region is believed to contribute to some of the above-cited less well-understood complications associated with VA ECMO. Based on this, an enhanced knowledge of the watershed zone behavior would be pivotal for optimizing the balance between the native and newly ECMO-driven oxygenation.

Investigating these aspects of VA ECMO remains challenging due to the intricate hemodynamic interactions involved. Both benchtop experiments (Schenk et al., 2015) and computational models (Cui et al., 2023) have been employed to study ECMO-related hemodynamics. Computational Fluid Dynamics (CFD) simulations have been extensively used in this field (Gramigna et al., 2024). Some studies have investigated the effect of ECMO support on arterial perfusion (Ahmed et al., 2022) and on the afterload increase (Vignali et al., 2023); while others have analyzed local fluid dynamics at the cannulation site (Parker et al., 2024). Additionally, prior numerical investigations have explored the watershed zone’s position and its variability at different ECMO flow rates (Rao et al., 2018). The morphology of the aorta is believed to play a key role in influencing the behavior of the watershed region, as highlighted in some previous publications (Khamooshi et al., 2024; Khodaee et al., 2022). Nevertheless, this statement is still to be proved, as tests on a wider number of cases are still lacking. In general, the state of the art most of the time reports single case studies (Du et al., 2022) or analyses performed on idealized aortic geometries (Malinowski et al., 2023). It is well established that the morphology of the thoracic aorta exhibits considerable anatomical variability, both in the main branch and in the supra-aortic vessels (Xiong et al., 2020), which may play a key role in ECMO-induced hemodynamic changes. In this research context, Statistical Shape Modeling (SSM) is emerging as a powerful tool for conducting population-based studies (Cosentino et al., 2020; Aljassam et al., 2024). SSM enables the extraction of key morphological features that characterize a population through two main steps: non-rigid registration of a template across all geometries of the dataset and dimensionality reduction to capture principal shape variations (Bruse et al., 2016). Moreover, the SSM is a tool that allows the data augmentation procedure, which means that it is possible to generate new realistic geometries (Ostendorf et al., 2024). The aim of this work is to conduct a population-based morphological and fluid dynamic study to investigate the effects of VA ECMO on flow distribution and blood mixing. Specifically, this study seeks to analyze the impact of aortic morphology on the watershed zone’s position and flow characteristics. We leveraged the capability of the SSM to expand our initial dataset with newly synthesized aortic geometries, whose morphology results from a combination of the original shapes. Then, a morphological analysis was performed to identify the most representative geometries within the augmented population. CFD simulations were then conducted on these selected geometries to assess how morphological differences influence hemodynamics. Based on the results of these initial simulations, a subset of geometries with the most significant hemodynamic features was selected for further analysis under ECMO-supported conditions. These additional simulations aimed to determine whether aortic morphology significantly impacts hemodynamics even in the presence of ECMO support. By integrating SSM and CFD, this study provides novel insights into the complex hemodynamics of VA ECMO, contributing to a better understanding of patient-specific factors that influence ECMO outcomes.

## 2 Materials and methods

**FIGURE 1:**
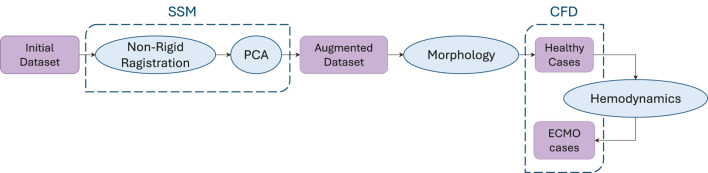
Workflow of the procedure followed in this study. Starting from an initial dataset, synthetic geometries were generated through SSM-based augmentation. A morphological analysis was then used to select geometries for healthy case simulations, and based on the hemodynamic analysis of these cases, a subset was chosen for ECMO-supported simulations.

The main steps of the procedure followed in this study are outlined in the following section and visually represented in Figure 1. Briefly, starting from an initial dataset of geometries, an augmented dataset of synthetic geometries was generated by developing an SSM. Through morphological inspection, the geometrically most representative geometries were selected for the healthy cases’ CFD simulations. Based on the hemodynamic analysis of these results, the most significant geometries were identified for simulations with ECMO support.

### 2.1 Dataset generation

This study considered an initial population consisting of 19 healthy thoracic aortas. The CT scans were performed with a 320-detector scanner (Toshiba Aquilion One, Toshiba, Japan) using an iodinated contrast medium. The images are characterized by an average pixel size of 0.625 mm and a slice thickness of 0.5 mm . Starting from this population, an SSM was developed. The non-rigid registration of a template on all the other target meshes of the dataset was achieved by using the novel algorithm described in Scarpolini et al. (2023). Given its central role, the template geometry was computed through an iterative procedure. Starting from an initial representative shape, point correspondences were optimized and updated iteratively until convergence was reached, defined as a Euclidean distance change below 0.1 mm. This algorithm enabled the inclusion of supra-aortic vessels in the analysis of the thoracic aorta by employing a multi-scale approach, coupled with the introduction of landmarks at the vessel’s open boundaries. Regarding the dimensionality reduction part, Principal Component Analysis (PCA) was applied, which allowed us to go from 19 meshes with 25.000 nodes to 19 meshes with 19 principal components, also called modes *m*. An augmented dataset of new realistic aortic geometries was generated by varying the standard deviation (SD) of each mode between ± 3. Since the modes are uncorrelated and ordered by decreasing variance, more geometries were retained for the first modes, using a unit step for the SD, compared to the later ones, for which only the change to +3 SD was maintained. This choice was driven by the goal of creating an augmented dataset with maximum heterogeneity, and keeping a uniform step size for the last modes did not result in significant geometric changes, as the explained variance is less than 1% .

### 2.2 Quantitative analysis

**FIGURE 2:**
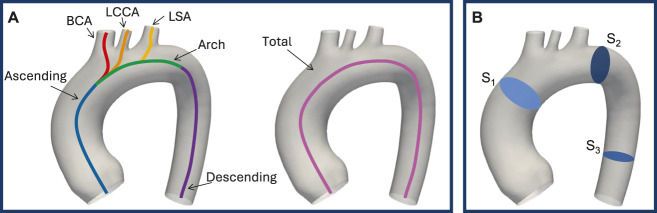
Seven characteristic tracts of the thoracic aorta: ascending aorta, arch, descending aorta, BCA, LCCA, LSA, and total aorta **(A)**. Result planes, in blue, on the template geometry **(B)**.

A quantitative analysis was conducted to evaluate the morphological differences between the generated shapes and the template. First of all, the centerlines of the vessels with the associated geometric characteristics were extracted through the VMTK software. This operation creates four poly-lines, each connecting the inlet at the aortic root to one of the four outlets: BCA, LCCA, LSA, and descending aorta. The seven characteristic tracts of the thoracic aorta (total aorta, ascending aorta, arch, descending aorta, BCA, LCCA, LSA), represented in Figure 2A, were computed starting from these poly-lines by applying the *branchextractor* operator coupled with an in-house script. The *branchextractor* separates each polyline into different tracts every time the maximum inscribed radius changes, so it was possible to obtain the seven segments working with the Centerline-Id and the Tract-Id. For each of the extracted parts (i) length, (ii) tortuosity: length of a line divided by the distance of its endpoints, (iii) maximum inscribed sphere radius (referred to as “radius” for the sake of brevity), (iv) curvature: inverse of the radius of the local osculating circle, and (v) torsion: the amount by which the osculating plane rotates along the line, were calculated. For points (iii) to (v) mean and standard deviation values were calculated. Finally, the difference of each quantity from the template value was calculated, enabling the selection of 20 geometries for the healthy simulations that accounted for the morphological changes induced by the modes. Moreover, the mean and standard deviation values were calculated for each quantity over all the centerlines to make a comparison with the template, which represents the average geometry of the population under study.

### 2.3 CFD simulations

**FIGURE 3:**
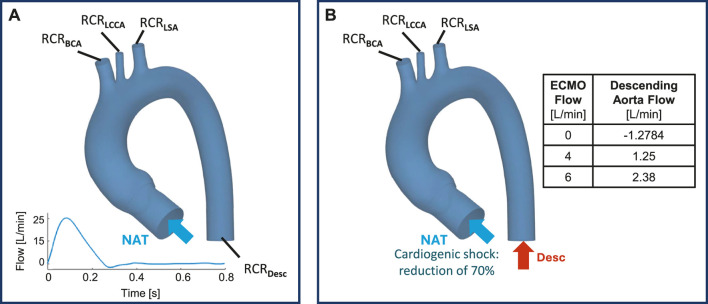
Simulations set up: healthy cases **(A)**; ECMO support **(B)**.

CFD simulations were performed to investigate how different aortic morphologies could impact the oxygenation of supra-aortic branches during extracorporeal membrane oxygenation. Specifically, a set of cases was chosen to simulate healthy conditions based on geometric criteria from the previous quantitative analysis. Following these, a subset of geometries was selected from the healthy cases to simulate ECMO-supported conditions based on differences in terms of mean flows. For all the simulations, the volumetric meshes were generated in ANSA BETA CAE with an automatic in-house script. A polyhedral mesh was adopted, characterized by an average element edge size of 0.5 mm and an imposed target skewness of 0.45. Simulations were performed in $Ansys^{TM}\;Fluent^{TM}$ (Ansys, Canonsburg, Pennsylvania, US) resolving the governing Navier-Stokes equations (Equation 1):

$$\begin{array}{c} \nabla \cdot \vec{v}=0\\ \frac{\partial \vec{v}}{\partial t}+\left(\vec{v}\cdot \nabla \right)\vec{v}=-\frac{1}{\rho }\nabla P+\nu \nabla^{2}\vec{v} \end{array}$$ (1)

where $\vec{v}$ is the fluid velocity vector, P is the fluid pressure, ρ the density of blood, and ν the kinematic viscosity of blood. The blood flow was modeled as an incompressible, Newtonian fluid in laminar condition with constant density ρ equal to 1060 $kg/m^{3}$ and viscosity ν equal to 3.5 $10^{-3}Pa\cdot s$ . Three cardiac cycles of 0.8 s each were simulated. At the outlet boundaries (BCA, LCCA, LSA, and descending aorta for the healthy simulations), a pressure condition was applied through a User Defined Function, implementing the three-element Windkessel model (Figure 3A), known as the RCR model. This model consists of a proximal $R_{p}$ in series with a parallel made of a distal $R_{d}$ resistance and a capacitance C . The relationships between flow rate Q(t) and pressure P(t) at each branch can be expressed, according to the RCR model, as reported in Equation 2:

$$P\left(t\right)=\left[P\left(0\right)-R_{\text{p}}Q\left(0\right)\right]e^{-t/\tau }+R_{\text{p}}Q\left(t\right)+\int_{0}^{t}\frac{e^{-\left(t-\bar{t}\right)/\tau }}{C}Q\left(\bar{t}\right)d\bar{t}$$ (2)

where $\tau =R_{d}C$ is the time constant that describes the system’s response speed to variations in the input function. For each outlet, the parameters were adjusted to maintain a physiological pressure range within the aortic domain and ensure an appropriate physiological flow split. The results were evaluated not only at the outlets but also in three additional sections: pre-arch, post-arch, and descending zone (Figure 2B). The planes for these sections were defined in Ansys Fluent using the 3-points method. The coordinates of these points were obtained through a fully automated process using a Python script that worked with the regions of the centerlines defined in Section 2.2. Based on the Point-Id, the points were identified at: (i) 85% of the ascending aorta for the pre-arch, (ii) 10% of the descending aorta for the post-arch, and (iii) 90% of the descending aorta. The coordinates and the Frenet tangent representing the normal to the point, perpendicular to the centerline, were used to define three planes, on each of which the 3-points are defined. To reduce simulation time, the process was automated using Python scripts and Journal files. The Journal file replicates the GUI command sequence, allowing $Ansys^{TM}\;Fluent^{TM}$ to configure input data and run calculations automatically. A single Journal file was used with a for loop to update the mesh path, velocity profile, and coordinate data for each aorta model. Simulations were run in batch mode via a Python script using the *Subprocess* module, which executed commands in the Windows command prompt while ensuring each simulation finished before starting the next.

### 2.4 Healthy simulations

For the healthy cases subset simulations, an inlet velocity condition was imposed at the aortic valve level. To investigate the impact of morphology, the same ideal flow rate waveform was used. Velocity values were scaled according to the aortic valve cross-section to impose the same flow (5 L/min ) value for all the selected geometries.

### 2.5 ECMO simulations

For the ECMO subset cases, a Mixture Model was adopted. The Mixture Model solves the momentum, continuity, and energy equations for the phases mixture while introducing the volume fraction for equations of the secondary phases, and algebraic expressions for the relative velocities (Stevens et al., 2017). A two-phase Mixture Model was used to simulate the presence of two fluids, where phase-1 represents the native blood entering from the ascending aorta, while phase-2 is blood from ECMO. Two inlet boundary conditions were set: (i) the pathological cardiac flow from the left ventricle at the aortic valve level and (ii) a constant level of ECMO flow (EF) at the descending aorta (Vignali et al., 2023) (Figure 3B). To simulate the cardiac shock condition, the velocity waveform imposed at the aortic valve level was scaled to obtain a 70 % reduction of cardiac output. Three levels of ECMO support were applied — 0, 4, and 6 L/min—resulting in three simulations for each mesh.

### 2.6 Evaluation parameters

For each simulated case, results were post-processed in Ansys Fluent, and the following aspects were evaluated: (i) contour plot of total pressure on the aortic wall at systolic peak; (ii) streamlines at systolic peak and contour plot of velocity at the defined planes and (iii) mean flows $\bar{Q}$ at the outlet, defined as reported in Equation 3:

$$\bar{Q}=\int Q_{i}\left(t\right)dt$$ (3)

For each CFD simulation, various output parameters were calculated.

## 3 Results

**FIGURE 4:**
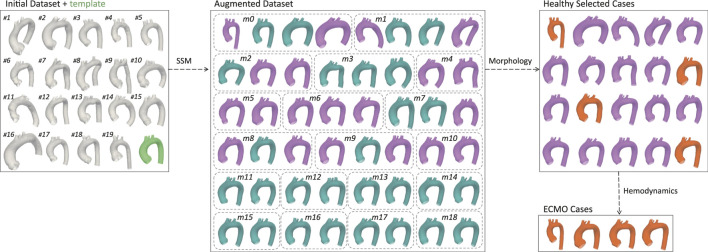
Graphical representation of the workflow. The initial dataset was augmented using SSM, generating an expanded set of geometries. Through morphological selection, a subset was defined for healthy simulations, and based on the hemodynamic results, specific geometries were selected for ECMO simulations. Labels have been added to identify the geometries from the initial dataset, and bounding boxes to group the augmented geometries according to their respective modes. In the augmented dataset the selected aortas for healthy simulations are colored in purple. In the healthy selected cases the aortas chosen for ECMO simulations are colored in orange.

Figure 4 presents the exploited graphical workflow outlining the methodology employed in this study. By developing an SSM, the initial dataset of 20 geometries was expanded to 48 synthetically generated, anatomically plausible geometries. A morphological analysis was then conducted to identify the 20 most representative geometries, which were subsequently used for CFD simulations under physiological conditions. Based on the hemodynamic analysis of these simulations, the four geometries exhibiting the greatest mean flow deviation from the template were selected for further CFD simulations under ECMO conditions.

### 3.1 Dataset generation

**FIGURE 5:**
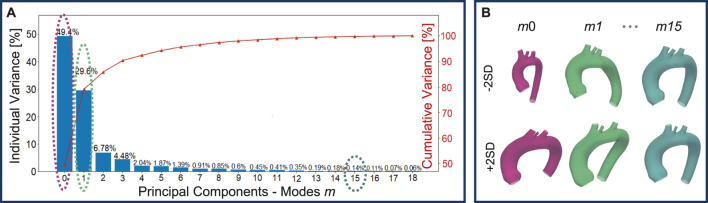
Individual and cumulative variance of the 19 principal components extracted through the PCA: as the modes increase, the percentage variance explained by each mode decreases **(A)**. Newly generated geometries by varying *m*0, *m*1, and *m*15 of ±2SD **(B)**.

A total of 48 geometries (47 novel + template) were generated through the SSM by varying the standard deviation of the PCA modes. A qualitative analysis, conducted through visual inspection of the generated aortic shapes, allows for an understanding of the morphological changes associated with each principal mode. In particular, Figure 5A, shows that the variance explained by each mode decreases as the mode index increases. As a result, the first modes, which explain the largest portion of the variance, are responsible for global shape variations and predominantly influence global features such as vessel length, curvature, and radius. Conversely, higher-order modes tend to produce more subtle deformations, especially in the supra-aortic branches, with progressively less noticeable effects on overall shape. For illustrative purposes, Figure 5B, depicts the morphological changes induced by modes *m*0, *m*1, and *m*15 as SD varies from −2 to +2.

### 3.2 Quantitative analysis

**FIGURE 6:**
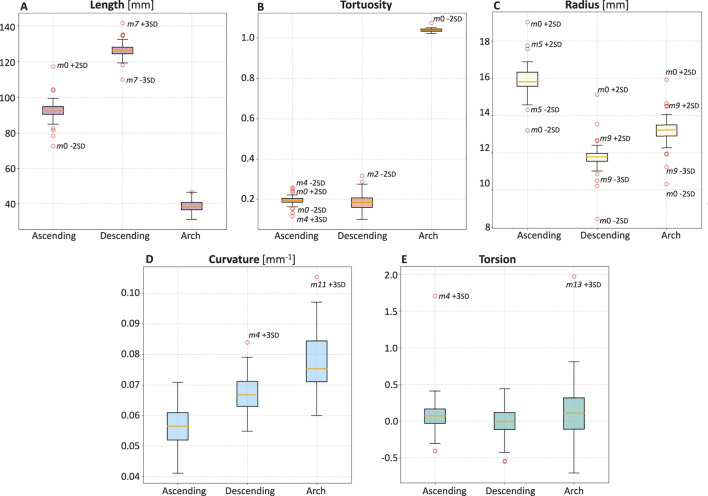
Boxplots of length **(A)**, tortuosity **(B)**, radius **(C)**, curvature **(D)**, and torsion **(E)** of the three main sections of the aorta: ascending, descending, and arch.

**FIGURE 7:**
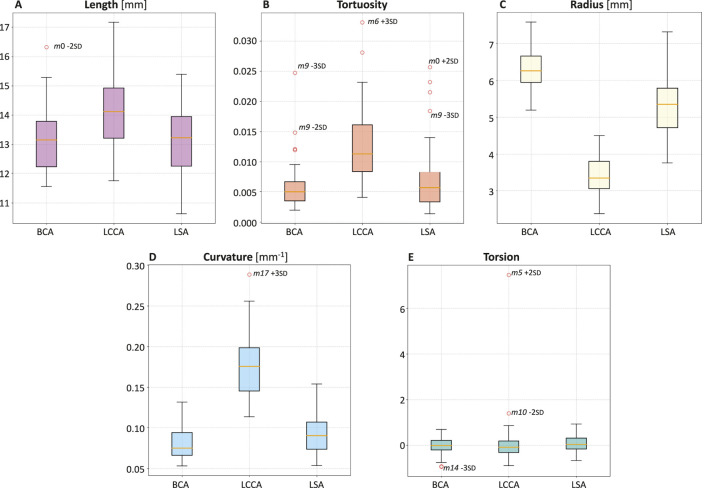
Boxplots of length **(A)**, tortuosity **(B)**, radius **(C)**, curvature **(D)**, and torsion **(E)** of the three supra-aortic branches: BCA, LCCA, LSA.

Subsequently, a quantitative analysis was conducted on the 47 aortas and the template to define the length, tortuosity, radius, curvature, and torsion of the three main sections of the aorta (ascending, descending, and arch tracts, Figure 6) and of the three supra-aortic branches (BCA, LCCA and LSA, Figure 7). The same features were also defined over the entire aorta to facilitate comparison of the various geometries. Modes 0 to three carry the majority of the variance as regards the length of the ascending, descending, and arch tracts. Extreme cases are visible for *m*0 and *m*7 for the ascending and descending tracts, respectively (Figure 6A). From mode 10 to 18, the total length variation is stable, around 2–3 % . The length of each tract was also compared to the corresponding tract of the template, and the lowest variability is in the descending tract.

Concerning tortuosity, most of the variability is observed in the descending aorta, while, in the ascending aorta, there are extreme cases reported by the variations of *m*0 and *m*4 (Figure 6B). From *m*10 to *m*18, there is less and stable variation. The tortuosity of the ascending and descending tracts, compared to the template, shows that the descending tract reaches higher variation.

Mode *m*0 carries most of the radius variance. The ascending tract presents the highest radius values, which decrease in the aortic arch and reach a minimum value in the descending zone (Figure 6C). Apart from *m*0, radius variations compared to the template for each tract show that the ascending radius has a comparable variation in *m*5 while the arch and descending radius in *m*9.

The curvature values are uniformly distributed between the different geometries, with values ranging from 0.04 to 0.10 $mm^{-1}$ (Figure 6D).

Torsion values are similar between the different geometries, with some extreme cases such as *m*4 +3 SD for the ascending tract and *m*13 + 3 SD for the arch (Figure 6E).

Table 1 reports the values of the morphological parameters. For each parameter, the values corresponding to the ascending aorta, descending aorta, and aortic arch are shown for the template geometry, along with the minimum and maximum values observed in both the initial dataset (composed of real aortic geometries) and the augmented dataset (synthetically generated shapes).

**TABLE 1:** Morphological parameter (Length [mm], Tortuosity, Radius [mm], Curvature $\left[mm{-}^{1}\right]$ , and Torsion) values for the template (Temp) and the most extreme geometries in both the initial (Init) and augmented (Aug) datasets. For each parameter, values are reported for the ascending aorta, descending aorta, and arch. The minimum and maximum values are reported with identifying labels in parentheses ( # number for real cases and shape mode with SD value for augmented cases).

Params	Region	Temp	Min-Init	Min-Aug	Max-Init	Max-Aug
Length	Asc	89.65	55.52 ( # 5)	72.61 (*m*0 -2)	104.37 ( # 16)	117.23 (*m*0 +2)
	Desc	124.3	112.72 ( # 13)	109.85 (*m*7 +3)	128.23 ( # 4)	141.73 (*m*7 -3)
	Arch	38.32	28.74 ( # 7)	31.28 (*m*0 -2)	60.15 ( # 8)	46.56 (*m*1 +2)
Tortuosity	Asc	0.18	0.08 ( # 5)	0.12 (*m*4 +3)	0.25 ( # 18)	0.26 (*m*8 +3)
	Desc	0.16	0.02 ( # 9)	0.1 (*m*0 -2)	0.39 ( # 6)	0.32 (*m*2 -2)
	Arch	1.04	1.02 ( # 2)	1.02 (*m*12–3)	1.12 ( # 5)	1.07 (*m*0 -2)
Radius	Asc	16.04	12.41 ( # 5)	13.21 (*m*0 -2)	21.54 ( # 16)	19.03 (*m*0 +2)
	Desc	11.68	8.9 ( # 19)	8.45 (*m*0 -2)	16.17 ( # 16)	15.14 (*m*0 +2)
	Arch	13.14	9.38 ( # 9)	10.32 (*m*0 -2)	17.62 ( # 16)	15.92 (*m*0 +2)
Curvature	Asc	0.17	0.1 ( # 16)	0.04 (*m*1 +1)	19.06 ( # 8)	0.07 (*m*14 + 3)
	Desc	0.17	0.13 ( # 1)	0.05 (*m*17 + 3)	1.8 ( # 9)	0.08 (*m*4 +3)
	Arch	0.1	0.14 ( # 10)	0.06 (*m*14–3)	5.68 ( # 11)	0.11 (*m*11 + 3)
Torsion	Asc	4.6	−4.51 ( # 1)	−0.41 (*m*5 +2)	66.93 ( # 13)	1.71 (*m*4 +3)
	Desc	0.31	−1.96 ( # 8)	−0.56 (*m*4 +3)	85.5 ( # 2)	0.44 (*m*10 + 2)
	Arch	−0.06	−32.5 ( # 11)	−0.71 (*m*7 -3)	3.09 ( # 4)	1.97 (*m*13 + 3)

Upon analyzing the differences between the supra-aortic branches, it was found that LCCA has the smallest radius (Figure 7C) and twice the curvature compared to BCA and LSA (Figure 7D).

### 3.3 Healthy simulations

From the 48 aortas created, 20 geometries were selected from the first 10 modes, as these accounted for 98.5 % of the total variability within the dataset. As described above, the principal morphological variations are in: (i) length (approximately 20 % for the first modes); (ii) tortuosity (approximately 50 % ) and (iii) radius (the largest variation is 20 % for the total tract of *m*0, followed by 10 % for the ascending tract of *m*5 and 10 % for the arch radius of *m*5 and *m*9). The supra-aortic variations were distributed all over the modes, and comparing the three branches, LCCA was half the size compared to BCA and LSA. The 20 selected geometries for the healthy cases are: *m*0 ± 2 SD, *m*1 ± 2 SD, *m*2 ± 1 SD, *m*4 -2/+3 SD, *m*5 ± 2 SD, *m*6 -2/+3 SD, *m*7 ± 3 SD, *m*2 ± 3 SD, *m*9 ± 2 SD, *m*10 ± 2 SD + the template.

**FIGURE 8:**
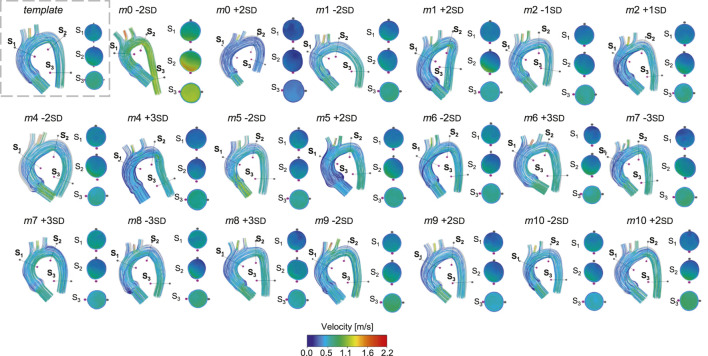
Velocity streamlines for the template and for the 20 geometries selected for the healthy simulations.

**FIGURE 9:**
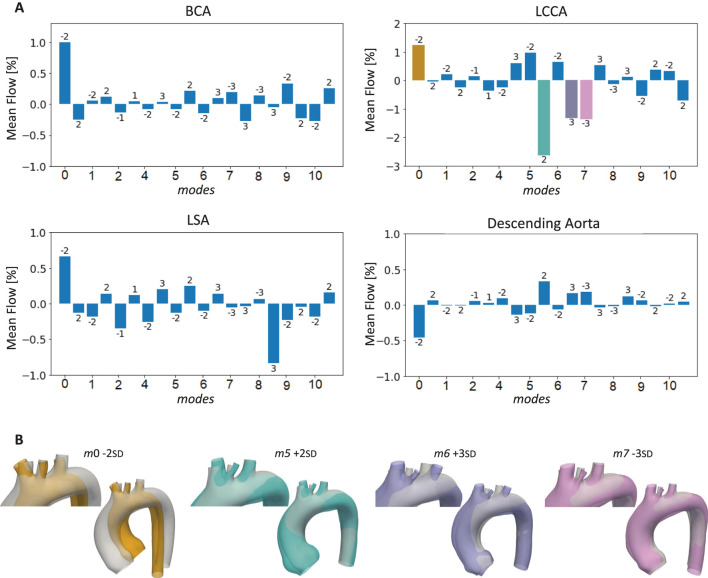
Bar plots of the mean flows at the outlets of the 20 aortas selected for the healthy simulations compared to the template **(A)**, with highlight on the cases with the largest LCCA mean flow variations **(B)**.

Pressure values across the 20 aortic geometries were compared to assess variations related to morphological changes. Overall, pressures remained consistent among all cases, with values ranging around 120–130 mmHg , and showing a physiological decrease from the ascending to the descending aorta. Figure 8 represents the streamlines for the simulated healthy cases. It is worth noting that *m*0 -2 SD and *m*1 +2 SD velocity reached the highest values. This phenomenon can be motivated by the arch shape and cross-section. In fact, the arch radii of *m*0 -2 SD and *m*1 +2 SD (10.32 mm and 12.49 mm , respectively) resulted to be lower in comparison with the template (13.14 mm ), while the tortuosities (4.63 and 3.29, respectively) appeared to be higher (2.78). This behavior can be associated with the arch shape (gothic arch Margarint et al. (2024); Marrocco-Trischitta et al. (2020)) in general, of these two given cases. Across all supra-aortic branches, LCCA had the highest velocity. This can be motivated by the fact that LCCA exhibited a reduced mean radius (3.4±0.5 mm) in comparison with BCA (6.3±0.47 mm) and LSA (5.3±0.79 mm) . Another morphological feature that influenced the streamlines was the common origin of BCA and LCCA, defined as the Bovine arch type. The geometries exhibiting these variations (*m*0 -2 SD and *m*1 +2SD) reached higher velocity values compared to the ones with the standard branching pattern. The bar plots in Figure 9 display the percentage of mean flow at the outlets (BCA, LCCA, LSA, and descending aorta) for all the healthy simulated cases with respect to the template mean flow. The largest mean flow variations were found in LCCA, in particular in *m*5 +2 SD, followed by *m*0 -2 SD, *m*6 +3 SD, and *m*7 -3 SD. Given this, the four above-cited cases were selected for the subsequent ECMO simulations.

### 3.4 ECMO simulations

**FIGURE 10:**
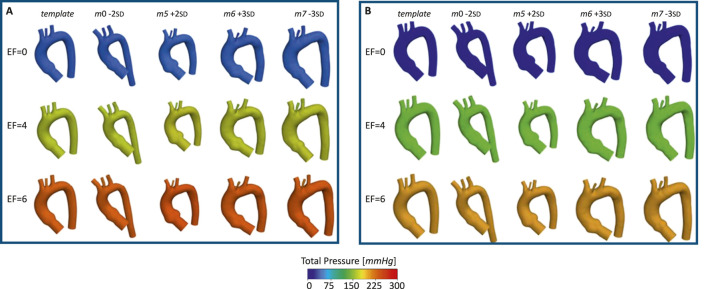
Total pressure for the template and the four aortas selected for the ECMO simulations, at different EF levels [L/min], for: systolic peak **(A)** and diastolic phase **(B)**.

**FIGURE 11:**
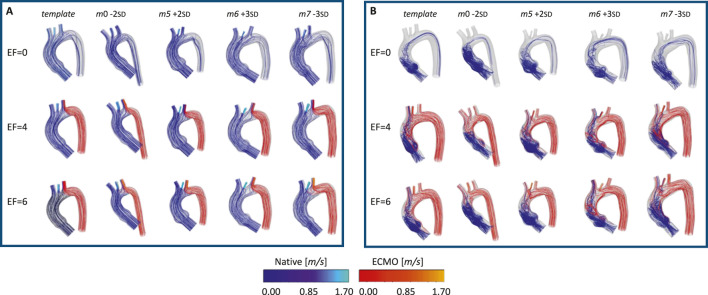
Velocity streamlines for the template and the four geometries selected for the ECMO simulations, at different EF levels [L/min], for: systolic **(A)** and diastolic phase **(B)**.

**FIGURE 12:**
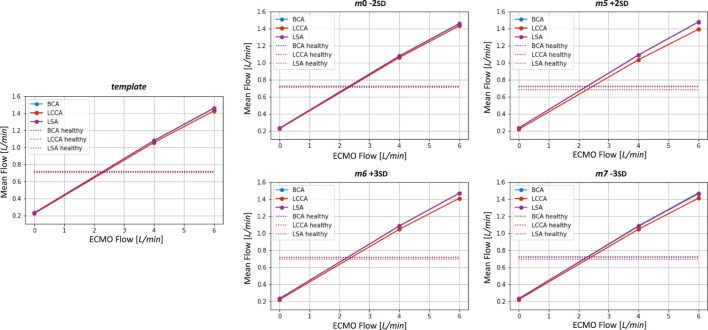
Mean flows as a function ECMO flow for the template and the four geometries selected for the ECMO simulations. Healthy levels of mean flow are also reported.

**FIGURE 13:**
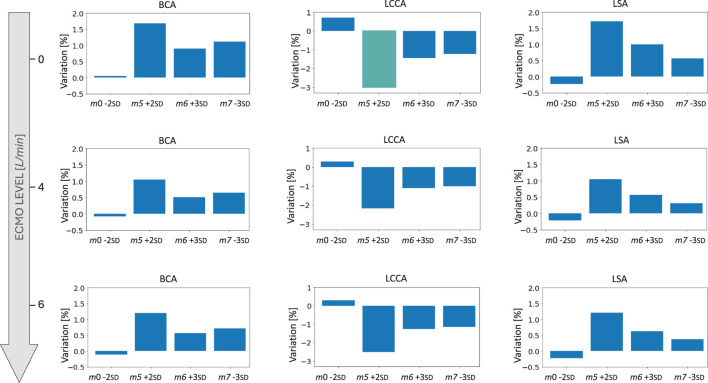
Bar plots of the mean flow percentage variations from the template for the four geometries selected for the ECMO simulations at different levels of ECMO support.

The pressures for the four simulated ECMO cases are reported in Figures 10A,B, for systolic peak and diastolic phase, respectively. In the absence of ECMO support, the simulated cardiogenic shock of 70 % caused a significant pressure drop, reaching a minimum value of approximately 50 mmHg . At 4 L/min ECMO support level, the pressure on the wall of the aorta during systolic peak exceeds the healthy value, reaching 160 mmHg . At a flow rate of 6 L/min, the pressure reaches even higher values, with peaks of 300 mmHg at systole. This behavior of pressure increase as a function of ECMO level is in accordance with the literature and the clinical evidence (Jiang et al., 2024; Gu et al., 2022; Yambe et al., 2024). By comparing the different geometries, the pressure values appear to be similar, and no significant difference in behavior emerged. The velocity streamlines for the four simulated ECMO cases are reported in Figures 11A,B, for the systolic peak and diastolic phase, respectively. For all cases, it was possible to qualitatively assess the native - ECMO flow mixing zone. Concerning the systolic peak (Figure 11A), at an ECMO level of 4 L/min, the mixing zone is located in the LSA at the systolic peak for all the four considered geometries. The increase of ECMO support level to 6 L/min causes the mixing zone to shift into the aortic arch, between LCCA and LSA. Concerning the diastolic phase (Figure 11B), it is possible to observe that the mixing always goes beyond the BCA level and reaches the ascending aorta both at 4 L/min and 6 L/min. By comparing different geometries, it is observed that the mixing zone position is not affected by morphological variations at the systolic peak, nor at diastole. Figure 12 shows the mean flow as a function of ECMO support levels for the three supra-aortic branches, with the healthy levels reported as reference. It is interesting to observe that the healthy level is always reached and the mean flow trend appears to be approximately linear in all the aortas, as already observed in previous works (Vignali et al., 2023). A quantitative comparison with the template was presented in the bar plots of Figure 13, where the mean flow percentage variations are represented. It is possible to observe that the differences in mean flows are limited, in the range between −3% and +2% . The maximum variation is found in the LCCA mean flow of *m*5 +2 SD, in which the flow encountered a reduction of approximately 3 % in the condition of absence of ECMO support. This behavior can be explained by the 20 % *m*5 +2 SD radius variation that emerged from the quantitative analysis. As can be seen from Figure 9B, the four geometries presented the Bovine arch type variation in the branching pattern, with the common origin of BCA and LCCA.

## 4 Discussion and conclusion

This study employed a novel combination of SSM and CFD to investigate the impact of aortic morphology on hemodynamics during VA ECMO support. The SSM approach successfully generated 48 different aortic morphologies, expanding the dataset beyond the initial patient-specific cases (Augmented Dataset of Figure 4). Figure 5 provides valuable insights into the role of principal component modes in capturing morphological variations. Panel A demonstrates that the variance explained by each mode decreases as the mode index increases. The ability of the first few modes to describe major structural changes suggests their potential in characterizing patient-specific aortic morphology for hemodynamic analysis. Additionally, Figure 5B highlights the morphological variations of three modes across a range of standard deviations. The variations of the first modes (*m*0 and *m*1) predominantly affect the global shape, influencing parameters such as aortic arch curvature and vessel diameter. In contrast, latter modes, specifically *m*15 in the example, introduce localized modifications and subtler changes, primarily affecting the supra-aortic vessels. Such local variations may influence secondary flow structures and localized hemodynamic stress distributions, which have been shown to play a role in vascular remodeling and pathology development (Li et al., 2023; Johnston et al., 2021). A quantitative morphology analysis was adopted to select a subset of cases for healthy and ECMO support simulations. The morphological characterization of the dataset revealed key variations in aortic segment properties such as length, tortuosity, radius, curvature, and torsion. Notably, modes 0 and seven exhibited the most extreme variations in length for the ascending and descending aorta, respectively (Figure 6A). Tortuosity varied significantly in the descending aorta (Figure 6B), which may have important implications for flow disturbances and the formation of secondary flow structures. The radius of the aorta showed the highest variation in the ascending portion (Figure 6C), which could affect local velocity profiles and pressure distributions. The results demonstrated that variations in aortic geometry influence the position and characteristics of the watershed zone, thereby affecting oxygenation distribution. These findings align with previous computational studies (Cheng et al., 2014; Khodaee et al., 2022), which have highlighted the role of anatomical variability in ECMO circulatory support. To evaluate the anatomical plausibility of the generated aortic geometries, we compared their morphological parameters with those of the real anatomies in the original dataset. Table 1 includes the minimum and maximum values observed for each parameter in both the initial and augmented datasets. The results show that the synthetic geometries lie within physiologically reasonable bounds, consistent with those found in real subjects. Slight deviations are observed for curvature and torsion, where the maximum values in the initial dataset are significantly higher than those in the augmented one. This is likely due to the lower variance explained by the higher-order modes used to generate the extreme synthetic shapes, where stronger morphological deviations would require higher SD values than the ± 2/ ± 3 range adopted in this study. This comparison supports the anatomical validity of the augmented dataset and highlights directions for future refinement.

Among the supra-aortic branches, the LCCA exhibited the smallest radius and the highest curvature values (Figures 7C,D), suggesting a more pronounced susceptibility to flow disturbances. The total pressure distribution remained relatively consistent across the 20 healthy simulations, with pressures around 120−130 mmHg in the ascending aorta, gradually decreasing in the descending aorta. This suggests that aortic morphological variations alone do not significantly alter systemic pressures under normal cardiac output conditions. However, localized pressure gradients in more tortuous geometries indicate potential areas of increased flow resistance. Flow streamlines in Figure 8 revealed distinct patterns influenced by arch morphology and branching configuration. In particular, geometries with increased tortuosity values (*m*0 and *m*4) exhibited higher velocity gradients and a more anteriorly positioned watershed zone. The mean flow distribution across supra-aortic branches showed substantial variations, particularly in LCCA (Figure 9A). Geometries featuring a bovine arch (Figure 9B) exhibited the largest deviations from the template, reinforcing the notion that anatomical variations at the arch level can significantly impact cerebral and upper-body perfusion. This corroborates findings from previous numerical studies indicating that complex aortic morphologies at the arch level may exacerbate flow disturbances and contribute to differential hypoxemia (Lo Russo et al., 2024; Syperek, 2020). Additionally, the presence of a bovine arch-type anatomy was associated with altered flow dynamics in supra-aortic branches, further emphasizing the role of anatomical variability in patient-specific ECMO outcomes.

The ECMO simulations demonstrated a marked increase in total pressure with higher ECMO flow rates (Figure 10). At 4 L/min, pressures exceeded physiological levels, reaching 160 mmHg, while at 6 L/min, localized peak pressures approached 300 mmHg. These elevations reinforce concerns regarding increased left ventricular afterload, which may compromise cardiac function and necessitate additional unloading strategies (Schrage et al., 2018; Distelmaier et al., 2020; Kishi, 2020; Jiang et al., 2024; Russo et al., 2019). The watershed zone position was highly dependent on ECMO support levels, as depicted in Figure 11. At 4 L/min, mixing predominantly occurred near the LSA, whereas at 6 L/min, the mixing region shifted into the aortic arch, between LCCA and LSA. During diastole, retrograde ECMO flow extended beyond the BCA, influencing the oxygenation distribution in the ascending aorta. Despite morphological variability, the watershed zone location remained relatively stable across different geometries. Mean flow distributions followed a largely linear trend with increasing ECMO support (Figure 12), suggesting a predictable redistribution pattern. Importantly, in all cases, healthy-level mean flow was restored, confirming ECMO’s role in maintaining systemic perfusion. However, cases with significant arch morphological deviations exhibited minor deviations in flow ratios, particularly in LCCA. The percentage variations in mean flow across different ECMO levels remained within −3% to +2% (Figure 13), with the most significant change observed in LCCA for *m*5 +2 SD. This reduction in LCCA flow may be linked to local geometric constraints, emphasizing the need for individualized ECMO planning, particularly in cases with altered arch anatomy (Ahmed et al., 2022). These findings are consistent with prior research (Nezami et al., 2021), which suggests that higher ECMO support levels may contribute to retrograde flow dominance, potentially leading to compromised oxygen delivery to cerebral and upper-body perfusion. The primary limitation of this study is the exclusion of the entire aortic morphology, as the model did not incorporate the abdominal aorta and iliac regions. This omission may influence the overall pressure and flow redistribution dynamics, particularly under ECMO support. Additionally, patient-specific boundary conditions were not included, which may limit the direct clinical applicability of the results. Moreover, although the computational model captures major hemodynamic trends, it inherently relies on assumptions and simplifications. Another important aspect, not included in the current scope of this study, is the characterization of helical flow structures. Helical flow in the aorta has been shown to play a protective physiological role by promoting smooth blood transport, stabilizing flow patterns, and reducing the formation of disturbed shear regions (Morbiducci et al., 2011; Gallo et al., 2012). Alterations in flow helicity have also been observed in pathological conditions, such as in patients with bicuspid aortic valves and aortic dilation, where the disruption of normal helical patterns may have clinical implications (Garcia et al., 2017). Quantifying helicity-related metrics in future simulations could thus enhance the understanding of flow dynamics under ECMO support and serve as a robust quantitative tool for assessing the mixing interface between native cardiac output and the ECMO jet. Additionally, it could be significant to evaluate the effect of the wall movement on the aortic hemodynamics (Capellini et al., 2018; 2021; Calò et al., 2023). Future studies should focus on expanding the dataset to include pathological aortic geometries, such as those encountered in atherosclerosis or aortic dissection, to further elucidate the role of vascular remodeling in ECMO hemodynamics.

An additional relevant aspect that could be explored in future work is whether the hemodynamic results obtained from the augmented dataset are consistent with those derived from the original patient-specific geometries. Such a comparison could serve as an additional validation of the fluid dynamic fidelity of the SSM-generated shapes. Nevertheless, it is worth noting that the augmented dataset was generated to preserve anatomical likeliness and geometric realism, as supported by the quantitative morphological analysis presented in this study. Additionally, incorporating validation through *in-vivo* measurements and experimental setups is fundamental to reinforce the credibility of numerical predictions. As recently demonstrated by (Gasparotti et al., 2025), who validated their CFD models using a detailed mock circulatory loop (Vignali et al., 2022; Bardi et al., 2022) combined with phantom-based flow measurements, the inclusion of experimental data provides an additional layer of verification. This dual numerical-experimental approach enhances the robustness of CFD simulations, enabling a more accurate characterization of flow patterns, critical mixing zones, and physiological responses under ECMO support. Future integration of such validation strategies will be essential to strengthen the reliability of computational frameworks and promote their adoption as effective clinical decision-support tools. Overall, this study highlights the critical role of aortic morphology in influencing ECMO hemodynamics. It provides valuable insights into the interplay between aortic morphology and ECMO hemodynamics, reinforcing the need for personalized ECMO strategies to optimize oxygenation and mitigate complications.

## Data Availability

The data that support the findings of this study are available on reasonable request from the corresponding author.
